# Supplementary material for: Practical and effective diagnosis of animal anthrax in endemic low-resource settings
Source: PLoS Negl Trop Dis. 2020 Sep 14;14(9):e0008655. doi: 10.1371/journal.pntd.0008655 (PMC7513992; doi:10.1371/journal.pntd.0008655)
Supplement: S1 Fig — (PDF) [file pntd.0008655.s001.pdf]

S 1 Fig. Chart used to establish presence and strength of *Bacillus anthracis* capsule material

<table>
<tr><td>+/-
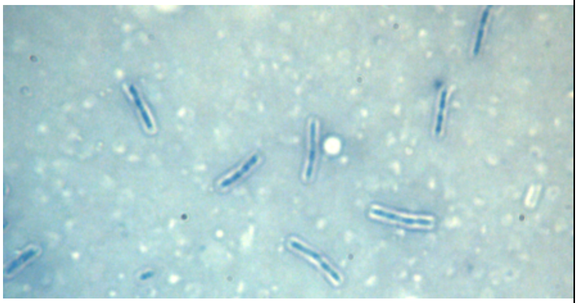
Presence of capsule well demarcated but not metachromatic. Stained with Giemsa</td><td>+/-
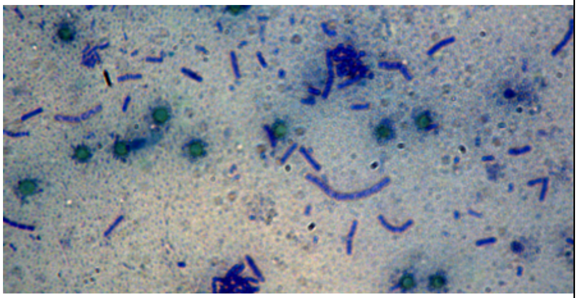
Pink outline faintly visible around the bacilli, but not demarcated. Stained with polychrome methylene blue</td></tr>
<tr><td>1+
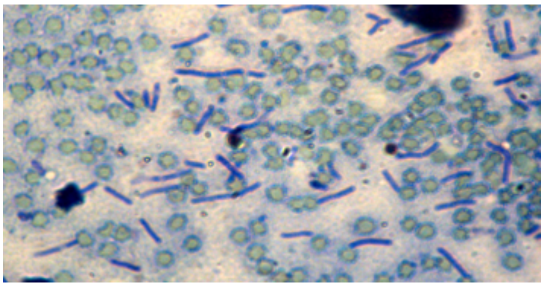
Capsule is visible as a faint pink weakly demarcated around the bacilli. Stained with azure B</td><td>2+
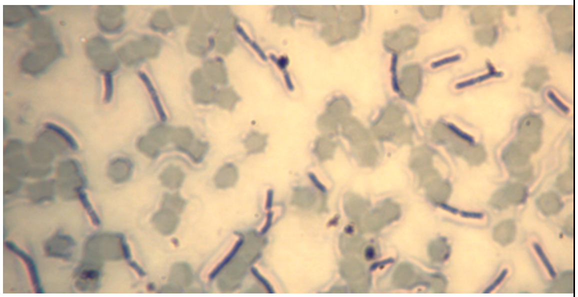
Capsule is moderately stained and well demarcated. Stained with Giemsa</td></tr>
<tr><td>3+
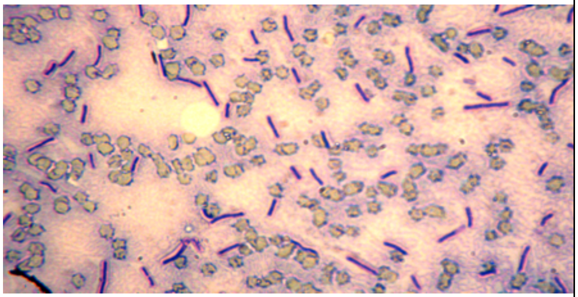
Capsule is strongly stained and well demarcated. Stained with azure B</td><td>-
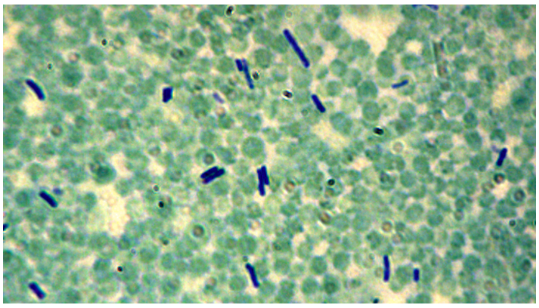
No evidence of capsule seen. Stained with polychrome methylene blue</td></tr>
</table>

The strength of the capsule is based on the presence of a demarcated capsule or a 'shadon' surrounding the cell and the metachromatic property of the capsule (Owen *et al.*, 2013). The chart is interpreted subjectively, and all categories (except those with – score) were interpreted as *B. anthracis* positive as they show evidence of the presence of a capsule. Samples with a +/- score are so indicated due to the absence of either a demarcated or metachromatic capsule. Images were obtained from pictures of slides examined in the study.

Reference

Owen, M. P. *et al.* (2013) 'A simple, reliable M'Fadyean stain for visualizing the Bacillus anthracis capsule.', *Journal of microbiological methods*, 92(3), pp. 264–9.
