# Supplementary material for: Practical and effective diagnosis of animal anthrax in endemic low-resource settings
Source: PLoS Negl Trop Dis. 2020 Sep 14;14(9):e0008655. doi: 10.1371/journal.pntd.0008655 (PMC7513992; doi:10.1371/journal.pntd.0008655)
Supplement: S1 Table — (PDF) [file pntd.0008655.s002.pdf]

**S 1 Table. Primer and probe sequences used in the qPCR reactions, targeting two plasmids and one chromosomal sequence in the *Bacillus anthracis* genome**

| Name of primer/probe | Sequence | Target | Reaction conditions |
|---|---|---|---|
| BA cap fwd* TM | GAA GCA GTA GCA CCA GTA AAA CAT C | pXO2 plasmid | 300 nM (0.6 µl of 10µM) |
| BA cap rev$^{\delta}$ TM | CTT TTA CGT GAC GTC CCA TCA | pXO2 plasmid | 900 nM (1.8 µl of 10µM) |
| BA cap prb$^{\gamma}$ TM | FAM TTG ACG ATG ACG ATG GTT GGT GAC A BHQ1 | pXO2 plasmid | 250 nM (0.5 µl of 10µM) |
| BA lef fwd* TM | GGA ACA AAA TAG CAA TGA GGT ACA AGA | pXO1 plasmid | 900 nM (1.8 µl of 10µM) |
| BA lef rev$^{\delta}$ TM | TTC CGG TGC ATA AAG CTG TAA AAC | pXO1 plasmid | 600 nM (1.2 µl of 10µM) |
| BA lef prb$^{\gamma}$ TM | FAM TTG CAT ATT ATA TCG AGC CAC AGC ATC GTG BHQ1 | pXO1 plasmid | 250 nM (0.5 µl of 10µM) |
| PL3_f* | AAAGCTACAAACTCT GAAATTTGTAAATTG | Chromosomal sequence | 250 nM (0.5 µl of 10µM) |
| PL3_r $^{\delta}$ | CAACGATGATTGGA GATAGAGTATTCTTT | Chromosomal sequence | 250 nM (0.5 µl of 10µM) |
| Tqpro_PL3 $^{\gamma}$ | FAMAACAGTACGTTT CACTGGAGCAAAAT CAABHQ1 | Chromosomal sequence | 150 nM (0.3 µl of 10µM) |

* Forward primer

$\delta$ Reverse primer

$\gamma$ Probe
