# Supplementary material for: Practical and effective diagnosis of animal anthrax in endemic low-resource settings
Source: PLoS Negl Trop Dis. 2020 Sep 14;14(9):e0008655. doi: 10.1371/journal.pntd.0008655 (PMC7513992; doi:10.1371/journal.pntd.0008655)
Supplement: S2 Table — (PDF) [file pntd.0008655.s003.pdf]

S 2 Table. Summary of samples and results for the 367 suspected anthrax cases investigated. Positive and negative samples are denoted with + and – respectively. PMB= polychrome methylene blue and NA implies no associated sample

ID	Method/Sample type								
	**Microscopy technique**				**Polymerase chain reaction**				
	Azure B	PMB	Giemsa	Rapi-Diff II	Blood smear	Whole blood	Blood swab	Skin	Flies
1	+	+	-	-	+	-	+	+	NA
2	+	+	-	-	+	+	+	+	NA
3	-	-	-	-	-	-	-	-	NA
4	-	-	-	-	-	-	-	-	NA
5	+	+	-	+	+	+	+	+	NA
6	+	+	-	-	+	+	+	+	NA
7	+	+	-	-	+	NA	+	+	NA
8	+	-	-	-	+	NA	+	+	NA
9	+	+	-	-	-	-	+	+	NA
10	-	-	-	-	+	-	+	-	NA
11	-	-	-	-	-	NA	+	-	NA
12	+	+	-	-	+	+	+	+	NA
13	+	+	-	-	+	+	+	+	NA
14	-	-	-	-	+	+	+	+	NA
15	+	+	-	-	+	NA	+	+	NA
16	+	+	-	+	+	+	+	+	NA
17	-	-	-	-	-	-	NA	-	NA
18	+	+	-	-	+	NA	+	+	NA
19	-	-	-	-	-	-	-	-	NA
20	+	+	-	-	+	NA	NA	+	NA
21	-	NA	-	-	-	-	-	-	NA
22	NA	NA	NA	NA	NA	NA	NA	-	NA
23	NA	NA	NA	NA	NA	NA	NA	-	NA
24	+	NA	+	-	+	+	+	+	NA
25	NA	NA	NA	NA	NA	+	+	+	-
26	+	NA	-	-	+	+	+	+	-
27	+	NA	-	-	+	-	+	+	NA
28	-	NA	-	-	-	-	-	-	-
29	-	-	-	-	-	-	-	-	NA
30	-	NA	-	-	-	NA	NA	NA	NA
31	NA	NA	NA	NA	NA	-	-	-	-
32	+	NA	-	-	+	+	+	+	NA
33	-	NA	-	-	-	-	-	-	-

ID	Method/Sample material								
	**Microscopy**				**Polymerase chain reaction**				
	Azure B	PMB	Giemsa	Rapi-Diff II	Blood smear	Whole blood	Blood swab	Skin	Flies
34	+	NA	-	-	+	+	+	+	-
35	NA	NA	NA	NA	NA	NA	NA	-	NA
36	NA	NA	NA	NA	NA	NA	NA	-	NA
37	-	NA	-	-	-	NA	NA	-	NA
38	+	+	+	-	+	NA	NA	NA	NA
39	-	NA	-	-	-	-	-	-	NA
40	+	+	-	-	+	NA	NA	NA	NA
41	-	NA	-	-	-	NA	NA	+	-
42	+	NA	-	-	+	NA	NA	NA	NA
43	-	NA	-	-	-	NA	NA	NA	NA
44	-	NA	-	-	NA	NA	NA	NA	NA
45	NA	NA	NA	NA	-	NA	NA	NA	NA
46	NA	NA	NA	NA	NA	NA	NA	+	NA
47	NA	NA	NA	NA	NA	NA	NA	+	NA
48	NA	NA	NA	NA	NA	NA	NA	+	NA
49	NA	NA	NA	NA	NA	NA	NA	NA	NA
50	-	NA	-	-	-	NA	NA	NA	NA
51	NA	NA	NA	NA	NA	NA	NA	NA	-
52	NA	NA	NA	NA	NA	NA	NA	NA	-
53	NA	NA	NA	NA	NA	NA	NA	NA	-
54	NA	NA	NA	NA	NA	NA	NA	NA	+
55	NA	NA	NA	NA	NA	NA	NA	NA	-
56	+	+	-	-	+	NA	NA	NA	NA
57	NA	NA	NA	NA	NA	NA	NA	-	NA
58	NA	NA	NA	NA	NA	NA	NA	+	NA
59	NA	NA	NA	NA	NA	NA	NA	+	NA
60	NA	NA	NA	NA	NA	NA	NA	+	NA
61	NA	NA	NA	NA	NA	NA	-	+	NA
62	NA	NA	NA	NA	NA	NA	NA	+	NA
63	NA	NA	NA	NA	NA	NA	NA	-	NA
64	NA	NA	NA	NA	NA	NA	NA	+	NA
65	NA	NA	NA	NA	NA	NA	NA	-	NA
66	NA	NA	NA	NA	NA	NA	NA	+	NA
67	NA	NA	NA	NA	NA	NA	NA	+	NA
68	NA	NA	NA	NA	NA	NA	NA	+	NA
69	NA	NA	NA	NA	NA	NA	NA	-	NA
70	+	+	-	-	+	+	+	+	NA
71	NA	NA	NA	NA	NA	NA	NA	+	NA

ID	Method/Sample material								
	**Microscopy**				**Polymerase chain reaction**				
	Azure B	PMB	Giemsa	Rapi-Diff II	Blood smear	Whole blood	Blood swab	Skin	Flies
72	+	+	+	+	+	NA	+	+	NA
73	-	-	-	-	-	NA	-	-	NA
74	-	-	-	-	-	NA	NA	NA	NA
75	+	NA	-	-	+	NA	NA	NA	NA
76	-	NA	-	-	-	NA	NA	NA	NA
77	-	-	-	-	-	-	+	-	NA
78	+	+	-	-	+	NA	+	-	NA
79	+	+	-	-	+	+	+	+	NA
80	+	+	-	-	+	+	+	+	NA
81	+	+	-	-	+	-	+	+	NA
82	-	-	-	-	+	NA	-	+	NA
83	-	-	-	-	-	+	-	-	NA
84	+	+	-	-	+	NA	+	+	NA
85	+	+	+	-	+	+	+	+	NA
86	+	+	-	-	+	NA	-	+	NA
87	-	-	-	-	-	-	-	-	NA
88	+	+	-	-	+	+	+	+	NA
89	+	-	-	-	+	+	+	+	NA
90	+	+	-	+	+	+	+	+	NA
91	+	+	-	-	+	+	+	+	NA
92	+	+	-	-	+	+	+	+	NA
93	NA	NA	NA	NA	NA	NA	+	+	NA
94	NA	NA	NA	NA	NA	NA	NA	+	NA
95	NA	NA	NA	NA	NA	NA	NA	+	NA
96	NA	NA	NA	NA	NA	NA	NA	-	NA
97	NA	NA	NA	NA	NA	NA	NA	-	NA
98	NA	NA	NA	NA	NA	NA	-	-	-
99	NA	NA	NA	NA	NA	NA	NA	+	NA
100	NA	NA	NA	NA	NA	NA	-	-	NA
101	NA	NA	NA	NA	NA	NA	+	+	NA
102	NA	NA	NA	NA	NA	NA	NA	+	NA
103	NA	NA	NA	NA	NA	NA	NA	+	NA
104	NA	NA	NA	NA	NA	NA	NA	+	NA
105	NA	NA	NA	NA	NA	NA	+	-	NA
106	+	+	-	-	+	+	+	+	NA
107	NA	NA	NA	NA	NA	NA	NA	+	NA
108	NA	NA	NA	NA	NA	NA	NA	+	NA
109	NA	NA	NA	NA	NA	NA	NA	+	NA

ID	Method/Sample material								
	**Microscopy**				**Polymerase chain reaction**				
	Azure B	PMB	Giemsa	Rapi-Diff II	Blood smear	Whole blood	Blood swab	Skin	Flies
110	NA	NA	NA	NA	NA	NA	NA	-	NA
111	NA	NA	NA	NA	NA	NA	NA	+	NA
112	-	-	-	-	-	-	-	-	NA
113	NA	NA	NA	NA	NA	NA	+	+	NA
114	+	+	-	-	+	+	+	+	NA
115	+	+	-	-	+	+	+	+	NA
116	+	+	-	-	+	NA	+	+	NA
117	NA	NA	NA	NA	NA	NA	NA	+	NA
118	NA	NA	NA	NA	NA	NA	NA	+	NA
119	NA	NA	NA	NA	NA	NA	NA	+	NA
120	NA	NA	NA	NA	NA	NA	NA	+	NA
121	NA	NA	NA	NA	NA	NA	NA	+	NA
122	NA	NA	NA	NA	NA	NA	NA	+	NA
123	-	-	-	-	-	-	-	-	NA
124	NA	NA	NA	NA	NA	NA	NA	-	NA
125	NA	NA	NA	NA	NA	NA	NA	+	NA
126	NA	NA	NA	NA	NA	NA	NA	+	NA
127	NA	NA	NA	NA	NA	NA	+	+	NA
128	NA	NA	NA	NA	NA	NA	+	+	NA
129	NA	NA	NA	NA	NA	NA	NA	-	NA
130	NA	NA	NA	NA	NA	NA	NA	+	NA
131	NA	NA	NA	NA	NA	NA	NA	-	NA
132	NA	NA	NA	NA	NA	NA	NA	+	NA
133	NA	NA	NA	NA	NA	NA	NA	-	-
134	NA	NA	NA	NA	NA	NA	NA	-	NA
135	NA	NA	NA	NA	NA	NA	NA	+	NA
136	NA	NA	NA	NA	NA	NA	NA	-	NA
137	NA	NA	NA	NA	NA	NA	NA	+	NA
138	NA	NA	NA	NA	NA	NA	NA	+	NA
139	NA	NA	NA	NA	NA	NA	NA	+	NA
140	NA	NA	NA	NA	NA	NA	NA	+	NA
141	NA	NA	NA	NA	NA	NA	NA	-	NA
142	NA	NA	NA	NA	NA	NA	NA	+	NA
143	NA	NA	NA	NA	NA	NA	NA	-	NA
144	NA	NA	NA	NA	NA	NA	NA	-	NA
145	NA	NA	NA	NA	NA	NA	NA	+	NA
146	NA	NA	NA	NA	NA	NA	NA	+	NA
147	NA	NA	NA	NA	NA	NA	NA	+	NA

ID	Method/Sample material								
	**Microscopy**				**Polymerase chain reaction**				
	Azure B	PMB	Giemsa	Rapi-Diff II	Blood smear	Whole blood	Blood swab	Skin	Flies
148	NA	NA	NA	NA	NA	NA	NA	-	NA
149	NA	NA	NA	NA	NA	NA	+	+	NA
150	NA	NA	NA	NA	NA	NA	NA	-	NA
151	NA	NA	NA	NA	NA	NA	NA	-	NA
152	NA	NA	NA	NA	NA	NA	NA	+	NA
153	NA	NA	NA	NA	NA	NA	NA	-	NA
154	NA	NA	NA	NA	NA	NA	NA	-	NA
155	NA	NA	NA	NA	NA	NA	NA	+	NA
156	NA	NA	NA	NA	NA	NA	NA	-	NA
157	-	NA	-	-	-	-	-	-	-
158	-	NA	-	-	-	-	-	-	-
159	NA	NA	NA	NA	NA	NA	NA	-	NA
160	NA	NA	NA	NA	NA	+	+	+	-
161	+	NA	+	-	+	NA	NA	NA	NA
162	NA	NA	NA	NA	NA	+	+	+	NA
163	-	-	-	-	-	-	-	-	NA
164	+	+	-	-	+	+	+	+	NA
165	+	+	-	-	+	NA	+	+	NA
166	+	NA	-	-	+	+	+	+	-
167	-	-	-	-	-	-	-	-	NA
168	NA	NA	NA	NA	NA	NA	NA	+	NA
169	NA	NA	NA	NA	NA	NA	NA	+	NA
170	NA	NA	NA	NA	NA	NA	NA	+	NA
171	NA	NA	NA	NA	NA	-	-	-	NA
172	NA	NA	NA	NA	NA	NA	NA	+	NA
173	NA	NA	NA	NA	NA	NA	NA	+	NA
174	NA	NA	NA	NA	NA	NA	NA	+	NA
175	NA	NA	NA	NA	NA	NA	NA	+	NA
176	-	-	-	-	-	-	-	-	-
177	NA	NA	NA	NA	NA	NA	NA	+	NA
178	NA	NA	NA	NA	NA	NA	NA	+	NA
179	NA	NA	NA	NA	NA	NA	NA	+	NA
180	NA	NA	NA	NA	NA	NA	NA	+	NA
181	NA	NA	NA	NA	NA	NA	NA	+	NA
182	NA	NA	NA	NA	NA	NA	NA	+	NA
183	NA	NA	NA	NA	NA	NA	NA	+	NA
184	NA	NA	NA	NA	NA	NA	NA	+	NA
185	NA	NA	NA	NA	NA	NA	NA	+	NA

ID	Method/Sample material								
	**Microscopy**				**Polymerase chain reaction**				
	Azure B	PMB	Giemsa	Rapi-Diff	Blood smear	Whole blood	Blood swab	Skin	Flies
186	-	NA	-	-	-	-	-	-	NA
187	NA	NA	NA	NA	NA	NA	-	+	NA
188	-	+	-	+	+	NA	+	-	NA
189	NA	NA	NA	NA	NA	NA	NA	-	NA
190	NA	NA	NA	NA	NA	NA	NA	+	NA
191	NA	NA	NA	NA	NA	NA	NA	+	NA
192	-	-	-	-	-	-	-	-	NA
193	NA	NA	NA	NA	NA	NA	NA	+	NA
194	NA	NA	NA	NA	NA	NA	NA	+	NA
195	NA	NA	NA	NA	NA	NA	NA	-	NA
196	NA	NA	NA	NA	NA	+	+	+	NA
197	+	+	-	+	+	+	+	+	NA
198	+	+	-	-	+	+	+	+	NA
199	+	+	-	-	+	+	+	+	NA
200	+	+	-	-	+	+	+	+	NA
201	NA	NA	NA	NA	NA	NA	NA	+	NA
202	NA	NA	NA	NA	NA	NA	NA	+	NA
203	NA	NA	NA	NA	NA	NA	NA	+	NA
204	NA	NA	NA	NA	NA	NA	NA	+	NA
205	NA	NA	NA	NA	NA	NA	NA	+	NA
206	NA	NA	NA	NA	NA	NA	NA	+	NA
207	+	+	+	-	+	+	+	+	NA
208	NA	NA	NA	NA	NA	NA	NA	+	NA
209	NA	NA	NA	NA	NA	NA	NA	+	NA
210	NA	NA	NA	NA	NA	NA	NA	+	NA
211	NA	NA	NA	NA	NA	+	+	+	NA
212	NA	NA	NA	NA	NA	NA	NA	+	NA
213	+	+	-	-	+	+	-	+	NA
214	+	+	-	-	+	+	+	+	NA
215	+	+	+	-	+	NA	+	+	NA
216	NA	NA	NA	NA	NA	NA	NA	+	NA
217	+	+	-	-	+	-	+	+	NA
218	-	-	-	-	-	-	-	-	NA
219	NA	NA	NA	NA	NA	NA	NA	+	NA
220	NA	NA	NA	NA	NA	NA	NA	+	NA
221	+	+	+	-	+	+	+	+	NA
222	-	-	-	-	-	NA	-	-	NA
223	NA	NA	NA	NA	NA	NA	-	-	NA

ID	Method/Sample material								
	**Microscopy**				**Polymerase chain reaction**				
	Azure B	PMB	Giemsa	Rapi-Diff II	Blood smear	Whole blood	Blood swab	Skin	Flies
224	NA	NA	NA	NA	NA	NA	NA	+	NA
225	NA	NA	NA	NA	NA	NA	NA	+	NA
226	NA	NA	NA	NA	NA	NA	NA	-	NA
227	NA	NA	NA	NA	NA	NA	NA	+	NA
228	-	-	-	-	-	-	-	-	NA
229	NA	NA	NA	NA	NA	NA	NA	+	NA
230	NA	NA	NA	NA	NA	NA	NA	+	NA
231	NA	NA	NA	NA	NA	NA	NA	+	NA
232	-	-	-	-	-	-	-	-	NA
233	NA	NA	NA	NA	NA	NA	NA	+	NA
234	NA	NA	NA	NA	NA	NA	NA	+	NA
235	NA	NA	NA	NA	NA	NA	NA	+	NA
236	NA	NA	NA	NA	NA	NA	NA	+	NA
237	+	+	-	+	+	NA	+	-	NA
238	NA	NA	NA	NA	NA	NA	NA	+	NA
239	NA	NA	NA	NA	NA	NA	NA	+	NA
240	NA	NA	NA	NA	NA	NA	NA	+	NA
241	NA	NA	NA	NA	NA	NA	NA	+	NA
242	NA	NA	NA	NA	NA	NA	NA	+	NA
243	-	+	-	-	+	+	+	+	-
244	NA	NA	NA	NA	NA	NA	NA	+	NA
245	+	+	-	+	+	+	+	+	NA
246	NA	NA	NA	NA	NA	NA	NA	+	NA
247	NA	NA	NA	NA	NA	NA	NA	+	NA
248	NA	NA	NA	NA	NA	NA	NA	+	NA
249	NA	NA	NA	NA	NA	NA	NA	+	NA
250	NA	NA	NA	NA	NA	NA	+	+	NA
251	NA	NA	NA	NA	NA	NA	NA	-	NA
252	NA	NA	NA	NA	NA	NA	NA	+	NA
253	+	+	-	+	+	+	+	+	NA
254	+	+	-	-	+	+	+	+	+
255	-	-	-	-	-	-	NA	-	NA
256	-	-	-	-	-	NA	-	-	NA
257	-	-	-	-	-	+	-	-	NA
258	NA	NA	NA	NA	NA	NA	NA	+	NA
259	NA	NA	NA	NA	NA	NA	NA	+	NA
260	-	-	-	-	-	+	-	-	NA
261	NA	NA	NA	NA	NA	NA	NA	+	NA

ID	Method/Sample material								
	**Microscopy**				**Polymerase chain reaction**				
	Azure B	PMB	Giemsa	Rapi-Diff II	Blood smear	Whole blood	Blood swab	Skin	Flies
262	NA	NA	NA	NA	NA	NA	NA	+	NA
263	+	+	-	-	+	+	+	+	NA
264	NA	NA	NA	NA	NA	NA	NA	-	NA
265	+	+	-	+	+	+	+	+	NA
266	-	-	-	-	+	-	-	-	NA
267	+	+	-	-	+	+	+	+	NA
268	-	-	-	-	+	NA	-	-	NA
269	+	+	-	-	+	NA	-	+	NA
270	NA	NA	NA	NA	NA	NA	NA	+	NA
271	+	+	-	+	+	+	-	+	NA
272	NA	NA	NA	NA	NA	NA	+	+	NA
273	NA	NA	NA	NA	NA	NA	+	-	NA
274	NA	NA	NA	NA	NA	NA	NA	+	NA
275	-	NA	-	-	-	-	-	-	-
276	NA	NA	NA	NA	NA	NA	NA	-	NA
277	NA	NA	NA	NA	NA	NA	NA	-	NA
278	NA	NA	NA	NA	NA	NA	NA	+	NA
279	-	NA	-	-	-	NA	NA	NA	NA
280	-	NA	-	-	-	NA	NA	NA	NA
281	-	-	-	-	-	NA	NA	NA	NA
282	NA	NA	NA	NA	NA	NA	NA	+	NA
283	NA	NA	NA	NA	NA	NA	NA	-	NA
284	NA	NA	NA	NA	NA	NA	NA	-	NA
285	NA	NA	NA	NA	NA	NA	NA	+	NA
286	NA	NA	NA	NA	NA	NA	NA	+	NA
287	+	+	-	-	+	NA	+	+	NA
288	NA	NA	NA	NA	NA	NA	NA	+	NA
289	NA	NA	NA	NA	NA	NA	NA	+	NA
290	NA	NA	NA	NA	NA	NA	NA	+	NA
291	NA	NA	NA	NA	NA	NA	NA	+	NA
292	NA	NA	NA	NA	NA	NA	NA	+	NA
293	NA	NA	NA	NA	NA	NA	NA	+	NA
294	NA	NA	NA	NA	NA	NA	NA	+	NA
295	NA	NA	NA	NA	NA	-	+	+	NA
296	NA	NA	NA	NA	NA	NA	NA	+	NA
297	NA	NA	NA	NA	NA	NA	NA	+	NA
298	NA	NA	NA	NA	NA	NA	NA	+	NA
299	NA	NA	NA	NA	NA	NA	NA	+	NA

ID	Method/Sample material								
	**Microscopy**				**Polymerase chain reaction**				
	Azure B	PMB	Giemsa	Rapi-Diff	Blood smear	Whole blood	Blood swab	Skin	Flies
300	NA	NA	NA	NA	NA	NA	NA	-	NA
301	NA	NA	NA	NA	NA	NA	NA	+	NA
302	NA	NA	NA	NA	NA	NA	NA	+	NA
303	NA	NA	NA	NA	NA	NA	NA	+	NA
304	NA	NA	NA	NA	NA	NA	NA	+	NA
305	-	-	-	-	-	-	-	-	NA
306	NA	NA	NA	NA	NA	NA	NA	+	NA
307	NA	NA	NA	NA	NA	NA	NA	+	NA
308	NA	NA	NA	NA	NA	NA	NA	+	NA
309	NA	NA	NA	NA	NA	NA	NA	+	NA
310	NA	NA	NA	NA	NA	NA	NA	-	NA
311	NA	NA	NA	NA	NA	NA	NA	+	NA
312	NA	NA	NA	NA	NA	NA	NA	+	NA
313	NA	NA	NA	NA	NA	NA	NA	+	NA
314	NA	NA	NA	NA	NA	NA	NA	-	NA
315	-	NA	-	-	-	NA	-	-	-
316	-	NA	-	-	-	NA	NA	NA	NA
317	-	NA	-	-	-	NA	NA	NA	NA
318	-	NA	-	-	-	NA	NA	NA	NA
319	+	NA	-	+	+	NA	NA	NA	NA
320	+	NA	-	-	+	NA	NA	NA	NA
321	NA	NA	NA	NA	NA	NA	NA	+	NA
322	NA	NA	NA	NA	NA	NA	NA	+	NA
323	NA	NA	NA	NA	NA	NA	NA	+	NA
324	NA	NA	NA	NA	NA	NA	NA	+	NA
325	NA	NA	NA	NA	NA	NA	NA	-	NA
326	NA	NA	NA	NA	NA	NA	NA	-	NA
327	NA	NA	NA	NA	NA	NA	NA	+	NA
328	+	+	+	+	+	+	-	+	NA
329	+	+	+	-	+	+	+	+	NA
330	NA	NA	NA	NA	NA	NA	-	-	NA
331	-	-	-	-	-	-	-	-	NA
332	-	-	-	-	-	-	-	-	NA
333	+	+	+	-	+	+	+	+	NA
334	-	-	-	-	-	-	-	+	NA
335	-	-	-	-	-	-	-	-	NA
336	NA	NA	NA	NA	NA	NA	NA	-	NA
337	NA	NA	NA	NA	NA	+	+	+	NA

ID	Method/Sample material								
	**Microscopy**				**Polymerase chain reaction**				
	Azure B	PMB	Giemsa	Rapi-Diff II	Blood smear	Whole blood	Blood swab	Skin	Flies
338	+	NA	-	-	+	NA	NA	NA	NA
339	+	+	+	-	+	NA	NA	NA	NA
340	NA	NA	NA	NA	NA	+	-	-	-
341	+	NA	-	-	+	NA	NA	NA	NA
342	-	NA	-	-	-	NA	NA	NA	NA
343	+	NA	-	-	+	NA	NA	NA	NA
344	NA	NA	NA	NA	NA	+	+	+	+
345	NA	NA	NA	NA	NA	NA	NA	+	NA
346	+	NA	-	-	+	NA	NA	NA	NA
347	-	NA	-	-	-	NA	NA	-	NA
348	+	NA	-	+	+	NA	NA	NA	NA
349	+	NA	-	+	+	NA	NA	NA	NA
350	-	NA	-	-	-	NA	NA	NA	NA
351	NA	NA	NA	NA	NA	NA	NA	+	NA
352	NA	NA	NA	NA	NA	NA	NA	+	NA
353	NA	NA	NA	NA	NA	NA	NA	-	NA
354	+	+	-	-	+	+	+	+	-
355	+	+	+	-	+	+	+	+	-
356	-	NA	-	-	-	-	-	-	-
357	-	NA	+	-	+	+	+	+	-
358	+	NA	-	-	+	+	-	-	-
359	-	-	-	-	-	-	-	-	NA
360	-	-	-	-	-	-	-	-	NA
361	-	NA	-	-	+	NA	NA	NA	NA
362	-	NA	-	-	-	NA	NA	NA	NA
363	-	NA	-	-	-	NA	NA	NA	NA
364	NA	NA	NA	NA	NA	NA	NA	-	NA
365	-	NA	-	-	-	NA	NA	NA	NA
366	+	NA	-	-	+	NA	NA	NA	NA
367	-	NA	-	-	-	NA	NA	NA	NA
