# Supplementary material for: Practical and effective diagnosis of animal anthrax in endemic low-resource settings
Source: PLoS Negl Trop Dis. 2020 Sep 14;14(9):e0008655. doi: 10.1371/journal.pntd.0008655 (PMC7513992; doi:10.1371/journal.pntd.0008655)
Supplement: S3 Table — (PDF) [file pntd.0008655.s004.pdf]

S 3 Table: Characteristics of the animal carcasses suspected to have died from anthrax, from which samples were collected

Variable	Number of samples with data (%)	Number of samples with missing values (%)
**Species**	**345 (94.0)**	**22 (6.0)**
Cattle	34 (9.3)	
Goat	37 (10.1)	
Sheep	247 (67.3)	
Donkey	17 (4.6)	
Giraffe	3 (0.8)	
Antelope	1 (0.3)	
Wildebeest	2 (0.5)	
Zebra	3 (0.8)	
Elephant	1 (0.3)	
**Age**	**330 (89.9)**	**37 (10.1)**
Juvenile	38 (10.4)	
Sub-adult	75 (20.4)	
Adult	217 (59.1)	
**Sex**	**172 (46.9)**	**195 (53.1)**
Female	116 (31.6)	
Male	56 (15.3)	
**Body condition prior to death (livestock species only, n=335)**	**301 (89.9)**	**34 (10.1)**
Fat	162 (48.4)	
Normal	136 (40.6)	
Thin	3 (0.9)	
**Intactness of carcasses prior to sampling**	**315 (85.8)**	**52 (14.2)**
Intact carcass	20 (5.4)	
Open carcass	295 (80.4)	
