# Supplementary material for: Practical and effective diagnosis of animal anthrax in endemic low-resource settings
Source: PLoS Negl Trop Dis. 2020 Sep 14;14(9):e0008655. doi: 10.1371/journal.pntd.0008655 (PMC7513992; doi:10.1371/journal.pntd.0008655)
Supplement: S4 Table — (PDF) [file pntd.0008655.s005.pdf]

S 4 Table. Comparison of capsule scores obtained with polychrome methylene blue and azure B

Capsule strength	Number of samples using azure B stain (n= 102)	Number of samples using polychrome methylene blue stain (n= 102)
0	40	40
+/-	10	9
1+	14	23
2+	26	20
3+	12	10
