# Supplementary material for: Practical and effective diagnosis of animal anthrax in endemic low-resource settings
Source: PLoS Negl Trop Dis. 2020 Sep 14;14(9):e0008655. doi: 10.1371/journal.pntd.0008655 (PMC7513992; doi:10.1371/journal.pntd.0008655)
Supplement: S3 File — (PDF) [file pntd.0008655.s008.pdf]

S 3 File. Effect of sampling time on the sensitivity of smear microscopy for the detection of *Bacillus anthracis*.

False negative samples were assessed in order to identify any association between the sensitivity of smear microscopy and time sample was collected since the death of the animal. These samples were those with negative microscopy results but assigned a positive status by the latent class model. False negative smears results were not more likely to be obtained from carcasses sampled late.

Indication of duration since death when false negative samples were collected where data are available.

Stain	Time collected/ Number of false negative samples (total negative samples)		
	24hours	24-48 hours	48hours – 1 week
Azure B	5 (40)	1 (26)	0 (0)
PMB	3 (20)	1 (20)	0 (0)
Giemsa	54 (89)	12 (40)	2 (2)
Rapi-Diff II	53 (88)	16 (41)	2 (2)

Timing of collection of true positive smear samples where data are available.

Stain	Time collected/ Number of true positive samples (total positive samples)		
	24hours	24-48 hours	48hours – 1 week
Azure B	60 (60)	16 (17)	2 (2)
PMB	50 (50)	9 (10)	1 (1)
Giemsa	11(11)	3 (3)	0 (0)
Rapi-Diff II	12 (12)	1 (2)	0 (0)
