# Supplementary material for: Practical and effective diagnosis of animal anthrax in endemic low-resource settings
Source: PLoS Negl Trop Dis. 2020 Sep 14;14(9):e0008655. doi: 10.1371/journal.pntd.0008655 (PMC7513992; doi:10.1371/journal.pntd.0008655)
Supplement: Attachment — Submitted filename: Responses to reviewersV3.docx [file pntd.0008655.s009.docx]

**PLOS Neglected Tropical Diseases submission (PNTD-D-20-00252)**

**Responses to reviewers’ comments**

We thank the reviewers, as well as the editorial team at PLOS Neglected Tropical Diseases, for reviewing our manuscript "Practical and effective diagnosis of animal anthrax in endemic low-resource settings" and for providing critical feedback and comments to improve the paper. We are pleased that all reviewers recognise the value of our research towards improving the surveillance of animal anthrax in endemic low-income contexts. We appreciate their constructive feedback, which has helped us improve the manuscript. We have incorporated changes according to the recommendations provided. Responses and changes made to the manuscript are described below each comment in blue. Two versions of the manuscript are submitted with this document. One clean version, and another with tracked changes showing where text edits have been made.

**Comments and responses**

Both reviewers raise the question about the limitation of PCR facilities in developing countries - since there was no third reviewer, I also reviewed this manuscript and also had a similar question.  I also agreed with the reviewer who suggested that figure S2 might be useful in the main manuscript.

Response: We acknowledge the reviewers’ concern and recognise that PCR facilities are limited in developing countries, especially in local laboratories. However, such facilities are increasingly becoming available in most countries in intermediate and national laboratories. Based on our experience, there has been reasonable investment in purchasing the required equipment at least at central level. The challenge we are facing mainly relates to setting up and retaining the capacity for these laboratories to carry out the procedures (i.e. in terms of ensuring timely procurement of supplies and reagents, and of providing their personnel with specialised training) rather than lack of equipment. In an attempt to help overcome this challenge in northern Tanzania, personnel at the Kilimanjaro Clinical Research Institute, where this study was conducted, have been trained to carry out PCR to detect *Bacillus anthracis*.

Our ability to conduct the laboratory work in Tanzania demonstrates that this type of work is possible in low-income countries. Beyond Tanzania, we are aware that laboratory infrastructure of adequate standard to conduct molecular-based diagnostics, as well as trained laboratory staff, are available across eastern Africa, for example in Kenya and Uganda. There is a concerted effort internationally to encourage the development of in-country molecular diagnostic capacity for anthrax confirmation in endemic settings, for example as part of the recommended strategy of the US Centers for Disease Control and Prevention (Vieira *et al*., 2017). We are therefore confident that this capacity will become increasingly widespread in low-income countries, providing unprecedented opportunities for anthrax surveillance and research. We now discuss some of these issues in the manuscript (lines 523-532).

S 2 Fig. has been moved to the main manuscript (now Fig 3).

**Methods**

**Reviewer #1:** For the purposes of validating the method being investigate here (azure B), it would have been valuable to do culture diagnostics on the same samples. I realize this may not have been feasible in-country, but wonder if it would have been possible to ship the samples out for this analysis?

Response: We acknowledge that bacterial culture would have been useful. However, this was not possible in Tanzania, which necessitated our study as the reviewer points out. Selected samples were shipped to collaborators in South Africa for culture, primarily with the goal of generating high quality and quantity of DNA for whole genome sequencing to enable the development of culture-independent genotyping methods for areas where culture cannot be carried out. Ultimately, we aimed to establish protocols that would enable these countries to set up and maintain their own diagnostics, removing the need for cumbersome and expensive transfer of samples to high-containment laboratories for culturing. There were very long delays of several months for obtaining import and export permits. With the storage conditions our samples were subjected to, the result was that many samples had growth of other organisms that precluded the growth of *B. anthracis* in PCR-positive samples (e.g. fungi, other spore-forming bacteria), even when using selective media. These specific conditions meant that culture had lower sensitivity than what would have normally been expected, and would have been a poor ‘gold standard’ in this situation (Berg *et al*., 2006; WHO, 2008). We made the most of the existing in-country infrastructure and given that we have applied recognised analytical tools to assess the tests in the absence of a ‘gold standard’, we do believe our assessment of the tests is robust.

**Reviewer #2:** There are no major concerns about the methods.

Just three questions:

1.) The authors define "suspected cases of anthrax" (lines 182-185). Does this definition also apply for older carcasses where only skin or pieces of skin were available? For livestock, the owners might remember the circumstances of death, but what about wild animals which were found dead and probably widely decomposed? How can anthrax be assumed as cause of death in these cases?

Response: wildlife represented a very small proportion of our samples (2.9%). These were opportunistic samples that were collected 1) from wildlife that were sufficiently intact to confirm no evidence of starvation or predation as the causes of death and 2) in situations where several wildlife deaths had occurred in an area within a short time span, suggestive of a disease outbreak. We have included a separate definition for wildlife cases by adding the following text in lines 198-201:

“We adapted this definition for wildlife to include carcasses 1) that were sufficiently intact to observe these signs and to preclude death from starvation or predation, and 2) in situations where several wildlife deaths had occurred in an area within a short span of time, suggestive of a disease outbreak.”

Generally, wildlife carcasses are often in too poor a condition to enable collection and testing of smears for microscopy. Our results, showing successful PCR on skin samples, are promising as they provide an alternative avenue for confirmation of wildlife cases which often go undetected/unconfirmed.

2.) The scores for staining are indicated in S1 Fig. Which staining method is shown in this Fig?

Response: We have stated the staining method for each of the images shown in S1 Fig

3.) Did the authors use a balance to determine the weight of the skin portions (50 mg) or was this just estimated?

Response: A balance was used to initially determine the weight of a few skin samples, providing us with an idea of the size of skin that would weigh approximately 50mg (this approximation is now indicated in the manuscript at line 279). Samples were not routinely weighed because it was not possible to move the available balance into the biosafety cabinet, and our risk assessment aimed to limit movement outside the biosafety cabinet.

**Results**

**Reviewer #1:** (No Response)

**Reviewer #2:** Results are clearly presented.

It would be interesting to know which PCR target was the most sensitive. From other publications, one could assume that this would be one of the plasmid targets because plasmids are present in more than one copy.

Response: We assume that targets with detectable florescence at lower cycle threshold (Ct) values are more sensitive than those with higher Ct values. In response to this comment, the text at lines 444-449 and 558-561 now reads:

“In many cases, florescence was detected for the *lef* target earliest (39.3% of samples where amplification of all three targets occurred) when compared to the *cap* (6.7%) and chromosomal targets (6.4%). For the other samples (52.4%) Ct values were the same either between two or all three targets. For the *lef* target, samples passed the Ct 1.6 cycles earlier on average compared to both *cap* and the chromosomal target *PL3*.”

“The higher analytical sensitivity of the *lef* target, which is carried by the PXO1 plasmid, is likely associated with the high number of copies of this plasmid typically present in the *B. anthracis* genome. The PXO2 plasmid which carries the *cap* target has been found to be present in much lower copy numbers [38]”

For completeness, a Table should be added to the supplementary material in which the results for each of the 367 suspected anthrax cases are summarized. This table will show the correlation between the PCR results for different sample materials of the same animal and also between staining and PCR of blood smears.

Response: A table has now been added to the supplementary materials (S 4 Table).

**Conclusions**

**Reviewer #1:** (No Response)
 **Reviewer #2:** The conclusions are supported by the data and limitations were discussed. The public health aspects were well covered.

Staining and PCR seem to be reliable methods for confirmation of anthrax in low-resource settings. However, DNA extraction using a homogeniser might not be available everywhere. Are there any alternatives for DNA extraction from skin samples without using beads?

Response: We carried out the homogenisation step because the equipment was readily available to us and we hypothesized that it might enhance the overall extraction efficiency. However, the DNeasy blood and tissue protocol does not call for this step and we believe that good results could still be obtained without homogenization. Unfortunately, it is not possible to quickly test the impact of including this step on the sensitivity in the present circumstances. Since this type of molecular testing is likely to be done at larger national-level facilities, such equipment may often be available. We have added some discussion to the manuscript at lines 569-572:

“While outside the scope of this study, further research would be valuable to assess whether simplified DNA extraction protocols from tissues (i.e. without the need of a homogeniser) would result in similar test accuracy and thereby minimise the amount of specialised equipment required.”

**Editorial and Data Presentation Modifications**

**Reviewer #1:** L80-83: It seems the problem of lack of infrastructure/consumables would also be a limitation for PCR. May need to add something here to strengthen the argument. Also, it seems the work here was conducted in a BSL3 facility (L248), so it does further raise the question about whether PCR based approaches alleviate the problem. The paper in general could use more discussion on previous use of PCR for anthrax diagnostics in clinical specimens (maybe not here, but in discussion).

Response: We have added some discussion of these issues to the manuscript as follows:

Lines 87-91 – “Besides the higher costs associated with culture, the occurrence of anthrax in very remote and challenging environments might mean that samples collected are not viable for culture when they eventually reach the laboratory, as *B. anthracis* is easily outcompeted by many other bacterial species [6].”

Lines 523-532- “Although our work was carried out in a BSL 3 laboratory, this is not a strict requirement for anthrax diagnostic procedures. Diagnostic procedures that do not generate aerosols or large quantities of the pathogen can be safely carried out in lower containment laboratories [6]. In the case of DNA extraction, this would ideally be done within a biosafety cabinet. There are ongoing concerted efforts to build the capacities of national laboratories in endemic countries to carry out molecular detection of *B. anthracis* from clinical specimens [37]. We therefore anticipate that this capacity will be widely available in low-income countries where anthrax is endemic, providing unprecedented opportunities for anthrax surveillance and research”.

L136: Possibly lead here by also indicating what is currently considered to be the “gold standard,” i.e., culture?

Response: The text at lines 140-145 now reads:

“Bacterial culture followed by confirmatory tests – including phage and penicillin sensitivity, and PCR to detect genes specific to B. anthracis – is currently considered the gold standard approach for the diagnosis of anthrax [6]. Although PCR may detect *B. anthracis* directly from samples where culture has been unsuccessful [22], neither direct PCR, which to our knowledge has rarely been implemented in field settings, nor microscopy are considered gold standard methods for the detection of *B. anthracis*.”

L157-158: Would it be possible to qualify the occurrence of anthrax in the NCA? For example, is it widespread and consistent, or does it occur sporadically? This would help support why the NCA may be a good system to validate the diagnostic approaches.

Response: We have added the following text at line 166-171

“Anthrax is present in the NCA and outbreaks are reported more frequently in the area in comparison to other regions [24,25]. Our ongoing active surveillance (since 2016) in the NCA indicates that the disease is much more widespread in humans and animals than reflected in official reports, with regular cases throughout the year. This provides an ideal setting for assessing the performance of anthrax diagnostic tests.”

L220: Indicate here that the samples used for PMB staining were a subset of the total samples.

Response: This has been indicated at line 236-237:

“PMB staining was carried out on a subset of the total samples (n=102) due to smear sample unavailability.”

L225, 281, 283: There seems to be a problem with the references here.

Response: The links have been corrected (now lines 241, 299, 301)

L347: Write out “NTC” rather than using acronym, since it only appears a couple times.

Response: We have written the acronym in full at all four appearances as suggested.

L366-367: What proportion of samples had all 5 sample types?

Response: Only 16 carcasses had all 5 sample types, representing 4.4% of the total cases investigated. This has now been indicated in the manuscript (lines 396&397)

“All five sample types were only obtained from 16 (4%) carcasses.”

L375-376: Does the time of sampling since death affect smear assay sensitivity? If approximate time since death is known, it may be helpful to assess whether there is a relationship between time since death and probability of being a false negative.

Response: The probability of being a false negative is the probability of being positive (based on model estimates) for those samples that are classified as negatives by the smear test. Linear regression modelling shows no significant association between the probability of a false negative and the time when the sample was collected since death, i.e. those collected between 24-48 h were no more likely to be a false negative (e.g. azure B; OR =1.00 CI95% (0.98 - 1.02) compared to those collected less than 24 h after death. In response to this, we have added the tables below as supplementary materials (S 3 File) and the following text to lines 405-408:

“The timing of smear collection was not associated with the sensitivity of the microscopy tests as samples collected more than 24 h after death were equally likely to be falsely negative as samples collected within 24 hours (for azure B, OR=1.00, 95% CI (0.98 – 1.02) (S 3 File).”

| Stain | Number of false negative samples/time collected | | |
| --- | --- | --- | --- |
|  | 24hrs | 24-48 hrs | 48hrs – 1 week |
| Azure B | 5 | 1 | 0 |
| PMB | 3 | 1 | 0 |
| Giemsa | 54 | 12 | 2 |
| Rapidiff | 53 | 16 | 2 |

For information- Indication of duration since death when true positive samples were collected

| Stain | Number of true positive samples/time collected | | |
| --- | --- | --- | --- |
|  | 24hrs | 24-48 hrs | 48hrs – 1 week |
| Azure B | 60 | 16 | 2 |
| PMB | 50 | 9 | 1 |
| Giemsa | 11 | 3 | 0 |
| Rapidiff | 12 | 1 | 0 |

L407-409: This section could use a sentence that highlights which sample type resulted in a qPCR assay with the highest sensitivity/specificity. Also, change “very high” to “high” (L407).

Response: These have been addressed (now at lines 454-456). The following text have been added:

“Quantitative PCR using smear samples yielded the highest combined sensitivity and specificity. For all sample types, the sensitivity and specificity of qPCR were high (87.0% - 98.6%) at the optimal sample-specific threshold (S4_Table)

L 446-449: It is not clear what level of observer experience is necessary to achieve the results presented here. This could use some discussion.

Response: We have added the following text

Lines 253-256 - “One of the observers, who had previous microscopy experience but had not been involved in routine *B. anthracis* diagnostics, was briefly trained to identify the morphological characteristics of this pathogen.”

Line 504 - “individuals – including those with minimal experience”

L491-500:  Perhaps this is stating the obvious, but it may be important to explain why fly samples have low sensitivity. For example, flies found in association with carcasses may not all have been exposed and infected with B. anthracis (so are not true positives), and here you are evaluating their potential as indicator species for the infection status of the nearby carcass.

Response: The reviewer is correct in that many of the flies might have had minimal contact time with the carcass, explaining why fly samples have such low sensitivity in comparison to other samples. We have added this clarification (lines 570-573):

“The potential to use flies as an indicator of the infection status of associated carcasses depends on the flies being exposed to *B. anthracis* from those carcasses, either through ingestion or as mechanical vectors [39]. Thus, flies not having picked up spores will falsely indicate an infected carcass as negative.”

Table 1: I might suggest renaming the column heading to be “Three stain comparison (n =152)” and “Four stain comparison (n=102)”. But, really, I’m not sure the first column adds that much more, so could be omitted.

Response: We appreciate the suggestion and think that this descriptive table provides further clarity to the methods section ‘microscopy testing’. The column headings have been changed.

Table 2: Should it be 95% credible posterior intervals reported here?

Response: We thank the reviewer for highlighting this. It has been changed to ‘posterior 95% credible intervals’

S1 Fig: It would be helpful to include suspect samples that were determined to be negative, so with similar morphology, but lacking the pink capsule or demarcated rod ends.

Response: An image has been added to S1 Fig: showing an example of a smear classed negative.

S2 Fig: I found this Figure helpful to illustrate the approach used, as well as summarize the various methods being assessed. You may want to consider moving this into the main text. Also, would it be possible to breakdown the model into its component classes (e.g., positive/ negative classes)?

Response: This figure has been moved to the main text (now Fig. 3). We assume the reviewer is suggesting adding the breakdown of positive and negative cases to the figure. While we appreciate the suggestion, we feel this would make the figure very busy and also muddle the distinction between study design/methods and results.

**Reviewer #2:**

Some references or links seem to be missing (e.g. line 225)

Response: The link has been corrected (now line 241)

**Summary and General Comments**

**Reviewer #1:**

This study evaluates the performance of microscopic and molecular-based methods for anthrax diagnostics from field-collected livestock and wildlife carcass samples. The authors’ main objective was to assess the use of a modified blood smear assay (using the azure B stain), which is more accessible in developing countries than culture-based methods. They found that the azure B method had a high sensitivity and specificity in comparison to 3 alternative microscopic staining methods, including the reference standard PMB method. Previous studies have tested the azure B method in the lab (e.g., Owen et al. 2013), but this appears to be the first (?) study to validate its application and performance in the field. Another goal of this study was to evaluate a qPCR-based assay for detecting *Bacillus anthracis* from a number of carcass sample types. The paper is well written, study design is robust, and conclusions appear to be valid. Ideally, the authors could have
included culture data as the gold standard for estimating the sensitivity/specificity of these methods, but it appears there was not a facility in-country equipped for this (hence the need for this paper!). Also, it is not clear whether the study is novel with respect to the use of PCR diagnostics, which is already an accepted standard. Overall, these results provide support for the azure B blood smear method and has the potential to improve surveillance and disease control in developing countries that lack infrastructure and resources.

Response: We value the reviewer’s positive outlook of the work. The reviewer is correct that this is the first study that tests the performance of azure B on field-prepared smear samples. Owen *et al*. (2013) tested the method on a limited number of smears prepared from cultured isolates. Our work on azure B is particularly relevant to local settings/labs with inadequate infrastructure for PCR or culture. With regards to the novelty of the PCR-based assay, while PCR is an accepted standard, we have not been able to find any previous references to its use on DNA directly extracted from samples for anthrax diagnostics in endemic settings (added at Line 144). In our field site, we found it a particularly useful alternative where azure B microscopy testing was not possible due to factors that make smear samples impossible/difficult to obtain (i.e. due to carcass consumption by humans and scavenging activity by wildlife). While the use of PCR for diagnostics itself is not new, we would argue that its practical application to overcome the challenges encountered in poor, rural, remote and endemic areas, e.g. the high performance on samples stored at ambient temperatures (up to 47 °C for several months), is novel and relevant. Additional text has been added to the Discussion at lines 535-537:

“To our knowledge, this study is the first to demonstrate the value of qPCR on DNA extracted directly from samples for the diagnosis of animal anthrax without the need for prior culture.”

**Reviewer #2:**

Anthrax surveillance in endemic low-resource settings is a problem, and the data presented in this manuscript will be helpful to establish reliable methods for confirmation.

Response: The reviewer’s favourable overview of the research is appreciated.

**References**

Berg, T., Suddes, H., Morrice, G. and Hornitzky, M. (2006) Comparison of PCR, culture and microscopy of blood smears for the diagnosis of anthrax in sheep and cattle. *Letters in applied microbiology*, 43(2), pp. 181–6.

Vieira, A. R. *et al.* (2017) Enhancing surveillance and diagnostics in anthrax-endemic countries. *Emerging Infectious Diseases*, 23, pp. S147–S153.

WHO (2008) *Anthrax in humans and animals*. Available at: https://www.who.int/csr/resources/publications/AnthraxGuidelines2008/en/ (Accessed: 7 January 2016).
